# The Encystment-Related MicroRNAs and Its Regulation Molecular Mechanism in *Pseudourostyla cristata* Revealed by High Throughput Small RNA Sequencing

**DOI:** 10.3390/ijms21072309

**Published:** 2020-03-26

**Authors:** Nan Pan, Muhammad Zeeshan Bhatti, Haiyang Zhang, Bing Ni, Xinpeng Fan, Jiwu Chen

**Affiliations:** 1 School of Life Sciences, East China Normal University, Shanghai 200241, China; PannanECNU@163.com (N.P.); bni@bio.ecnu.edu.cn (B.N.); 2 Shanghai Key Laboratory of Regulatory Biology, Institute of Biomedical Sciences, School of Life Sciences, East China Normal University, Shanghai 200241, China; zeshan34@yahoo.com (M.Z.B.); 13262266069@163.com (H.Z.); 3 Department of Biological Sciences, National University of Medical Sciences, Rawalpindi 46000, Pakistan

**Keywords:** ciliated protozoa, *Pseudourostyla cristata*, dormant cyst, vegetative cell, differential expression miRNA, signaling pathway

## Abstract

MicroRNAs (miRNAs) regulate the expression of target genes in diverse cellular processes and play important roles in different physiological processes. However, little is known about the microRNAome (miRNAome) during encystment of ciliated protozoa. In the current study, we first investigated the differentially expressed miRNAs and relative signaling pathways participating in the transformation of vegetative cells into dormant cysts of *Pseudourostyla cristata* (*P. cristata*). A total of 1608 known miRNAs were found in the two libraries. There were 165 miRNAs with 1217 target miRNAs. The total number of differential miRNAs screened between vegetative cells and dormant cysts databases were 449 with *p* < 0.05 and |log2 fold changes| > 1. Among them, the upregulated and downregulated miRNAs were 243 and 206, respectively. Furthermore, Kyoto Encyclopedia of Genes and Genomes (KEGG) analysis revealed that some of the differentially expressed miRNAs were mainly associated with oxidative phosphorylation, two-component system, and biosynthesis of amino acids. Combining with our bioinformatics analyzes, some differentially expressed miRNAs including miR-143, miR-23b-3p, miR-28, and miR-744-5p participates in the encystment of *P. cristata*. Based on these findings, we propose a hypothetical signaling network of miRNAs regulating or promoting *P. cristata* encystment. This study shed new lights on the regulatory mechanisms of miRNAs in encystment of ciliated protozoa.

## 1. Introduction

Ciliated protozoa are unicellular eukaryotes which show a high level of cell differentiation and are distributed worldwide in diverse habitats. Ciliates are found at various trophic levels of a food chain, which makes them important for the proper functioning of an ecosystem [1]. Many ciliates adopt encystment as a survival strategy, i.e., the vegetative cells of ciliates can transform into dormant cysts in responding to harsh environmental conditions e.g., starvation, crowding, sudden changes in temperature, and lack of food [2]. Studies on the dormant cyst formation contribute to the understanding of the mechanism of eukaryotic cell morphogenesis [3]. Several early studies have reported the induction factors, physiological and biochemical changes in the process of ciliate encystment [4,5], and there were also reports that documented the changes in macronuclear DNA demethylation during encystment and the protein post-transcriptional modification of cyst dormancy [6]. Research used microarray to identify genes that were specifically up- or downregulated in the *Tetrahymena thermophila* life cycle [7]. In the recent decade, several scientists have developed new techniques and methods to study the molecular mechanism of encystment in ciliates. Chen et al. identified that several proteins were associated with encystment of *Euplotes encysticus* including type II cytoskeletal 1, calpain-like protein, formate acetyltransferase, alpha S1 casein, and cold-shock protein by 2D electrophoresis and MALDI-TOF MS techniques [8]. Gao et al. also identified proteins and genes in dormant cysts of *P. cristata* including fibrillarin-like rRNA methylase, ADP ribosylation factor, Rab12, and MAPK-related kinase by shotgun LC-MS/MS and scale quantitative real-time PCR (qRT-PCR) analysis [9]. Jiang et al. found AMPK, CaMK, ABCC4, ERK1/2, and PP1 were associated with the cyst formation of *Colpoda aspera* through transcriptomic techniques [10]. Our previous report found calcium, AMPK, FOXO, and a ubiquitin-mediated proteolysis signaling pathway jointly regulated the encystment of *P. cristata* [3]. However, to the best of our knowledge, there is no attention that has been paid to the function of miRNAs involved in the regulatory formation of ciliate cysts.

MiRNAs are endogenous, non-protein coding small RNAs that post-transcriptionally modulate global gene expression in eukaryotic cells and are highly conserved across species [11]. Regulation of gene expression by miRNAs is at the heart of an ever-increasing of biological pathways and is drawing increasing attention of biologists in different research fields [12].

The present study aims to investigate the miRNA regulation of ciliate encystment. For the first time, we provided the complete miRNA profiles of vegetative cells and dormant cysts and the molecular mechanism of the cyst formation of ciliated protozoa by small RNA sequencing in the Illumina high-throughput sequencing (HiSeq) X Ten system. In addition, bioinformatics techniques were used to analyze the significance of miRNAs associated with the encystment of *P. cristata* and miRNAs target genes. Furthermore, the Gene Ontology (GO) and Kyoto Encyclopedia of Genes and Genomes (KEGG) pathway analyses revealed that these differentially expressed miRNAs (DEMs) regulated the encystment processes via oxidative phosphorylation, two-component system, biosynthesis of amino acids, PI3K-AKT, MAPK signaling, etc. The genome-wide miRNA profile from the vegetative cells and dormant cysts of *P. cristata* in the current investigation will be not only the useful information in further elucidation of the molecular mechanisms of cyst formation of *P. cristata* but also a novel insight into miRNA regulation in the encystment of ciliated protozoa.

## 2. Results

### 2.1. Small RNA Populations Collected from P. cristata by HiSeq Technology

A total of 31,646,444 and 33,344,602 raw reads were generated from the vegetative cells (yy) and dormant cysts (bn) databases, respectively. After quality and length filter, adaptor sequences, heterogeneous sequences, and quality control, 30,368,742 and 28,853,904 clean reads were collected for the two libraries, respectively (Table 1).

Overall, 25,273,381 (83.22%) and 15,131,859 (52.44%) unique sequences were mapped onto the reference genome between the vegetative cells and dormant cysts databases, respectively. After mapping with Rfam database, the sequence annotation results of rRNA, snRNA, Cis-reg, tRNA, and other Rfam RNA are shown in Table S1.

**Figure 1:**
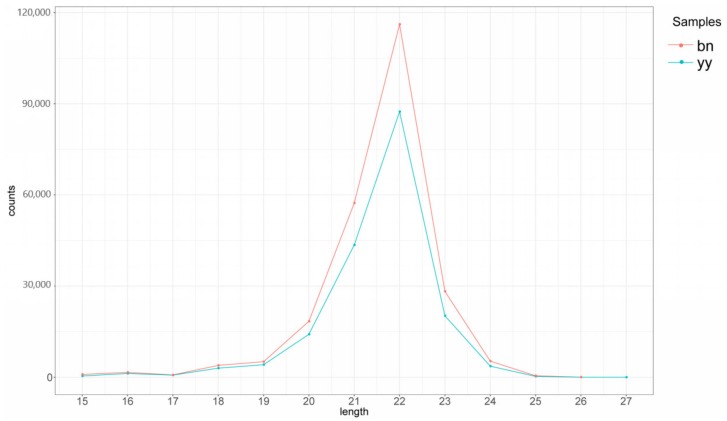
Known miRNA length distribution of *P. cristata*. Green represents vegetative cells (yy) and red indicates dormant cysts (bn).

Extracting sequence with a length of 15–26 nt by transcript sequence alignment used for subsequent known miRNA alignments and new miRNA predictions. The result of mRNA sequence and Repbase alignment statistics were represented in Tables S2 and S3, respectively. At last, the numbers of miRNA sequences were identified by comparing with the miRBase database (Table S4). In total, 1160 and 1271 known miRNA categories of the vegetative cells and dormant cysts database were identified based on the small RNA libraries (Table S5). In the two libraries, the majority of sequencing reads were between 19 and 22 nt in length. The known miRNA length distribution of vegetative cells and dormant cysts samples were mainly concentrated in 22 nt (Figure 1). In conclusion, the distributions of reads were compared on the databases of both samples (Table 2).

### 2.2. Identification Against Known and Novel miRNAs in P. cristata

The miRBase database analysis showed numbers of reads in the vegetative cells were 178,799 and dormant cysts were 238,353. Next, the expression of miRNA was counted and subsequent analysis represented 1608 known miRNAs in two libraries. Overall, 165 miRNAs targeted 1217 target genes. Furthermore, 1168 and 1277 miRNAs were detected in the vegetative cells and dormant cysts libraries, respectively. Among them, 1160 and 1271 were known miRNAs. These results showed that 837 miRNAs were co-expressed in all libraries, with 331 and 440 miRNAs exclusively expressed respectively in the vegetative cells and dormant cysts libraries. In addition, eight novel miRNAs were identified in the vegetative cells library and six novel miRNAs were recognized in the dormant cysts library using miRDeep2 software and RNAfold software (Table 3). In addition, all of the miRNAs identified in this study were classified into specific families based on their shared sequence similarity. Among these miRNAs, they were annotated to 227 and 239 families in the vegetative cells and dormant cysts libraries, respectively.

### 2.3. Universally Abundant miRNAs Across the Two Libraries

The miRNA plots of the known miRNA lengths detected for each sample were consistent with the miRNA trends of the vegetative cells and dormant cysts databases, and the length was mainly distributed at 22 nt. The known miRNAs showed a very broad range of expression that changed from fewer than 10 sequence reads to almost ten thousand sequence reads, which suggested that the majority of abundant miRNAs were from a few miRNA species.

The abundant miRNAs mainly belonged to the top 30 miRNA gene families, including let-7, mir-10, mir-30, etc., and some miRNAs dominated the miRNA libraries. For example, there were 84, 72, and 51 mature miRNAs annotated into the mir-10, let-7 and mir-30 families, respectively. In the unified set of the top 20 miRNAs over the two libraries, 9 (miR-26c, let-7a, let-7f and miR-21-5p, etc.) had the highest abundance in both libraries. In particular, miR-26c was the most abundant miRNA, where its sequence counts in the vegetative cells and dormant cysts libraries were 14,648 and 20,142, respectively. The top 20 abundant miRNAs and the top 30 miRNA gene families of the vegetative cells and dormant cysts databases were shown in Table S6 and Table S7, respectively.

### 2.4. Analysis and Validation of DEMs

**Figure 2:**
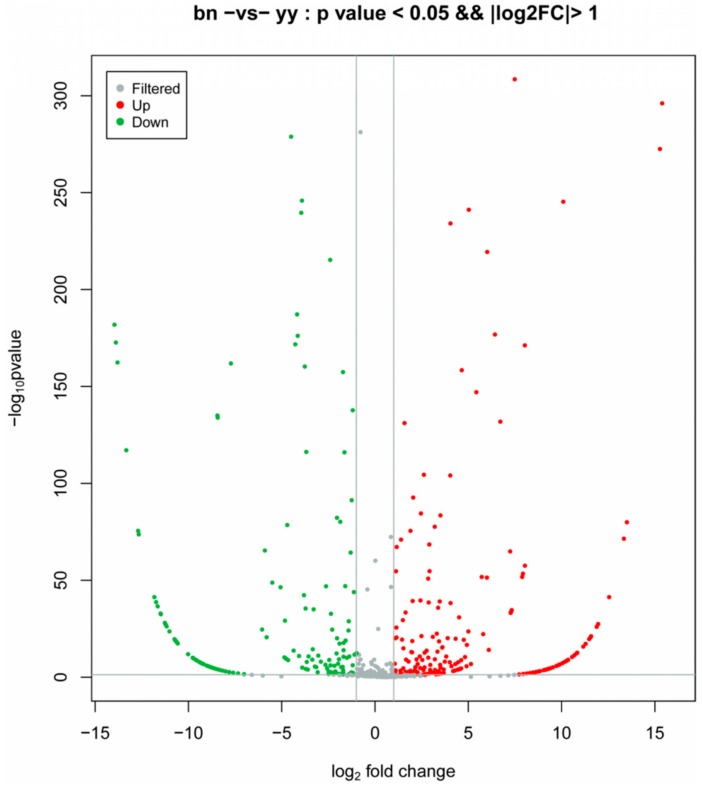
Volcano plot of DEMs showing the similarities and differences between vegetative cells (yy) and dormant cysts (bn) of *P. cristata*. Each point/dot in the diagram represents a DEM. The red dots represented upregulation, whereas green dots indicated downregulation of DEMs. Gray dots represented DEMs that were not differentially expressed. The *x*-axis displayed the log2 fold change, and the *y*-axis direction displayed the log10 *p*-value.

**Figure 3:**
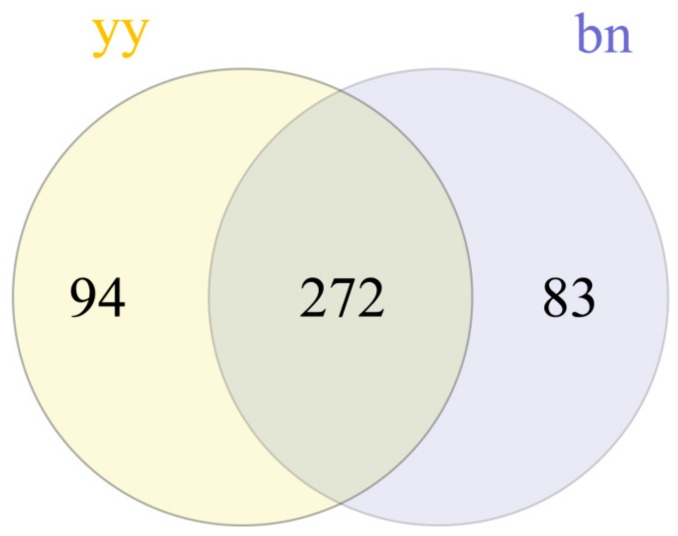
The Venn diagram showing the similarities and differences in DEMs between vegetative cells (yy) and dormant cysts (bn) of *P. cristata*.

The sequencing reads of 1608 known and eight novel miRNAs were counted in the samples of the vegetative cells and dormant cysts of *P. cristata*, and they were normalized using the transcript per million (TPM) values. The distribution of the TPM values showed that the miRNAs expression pattern differed significantly among the encystment of *P. cristata*. Among these, we identified a total of 449 DEMs between the two samples with *p* < 0.05 and |log2 fold changes| > 1. Whereas 243 DEMs were upregulated, while 206 DEMs were downregulated. We made a volcano map to further understand the overall distribution of DEMs (Figure 2). Compared to all DEMs, 272 DEMs were co-expressed in both groups. In addition, 94 and 83 DEMs were uniquely expressed in the vegetative cells and the dormant cysts groups, respectively. For an intuitive overview of these DEMs, a Venn diagram was constructed, which intuitively showed that DEMs varied between the dormant cysts and the vegetative cells (Figure 3).

**Figure 4:**
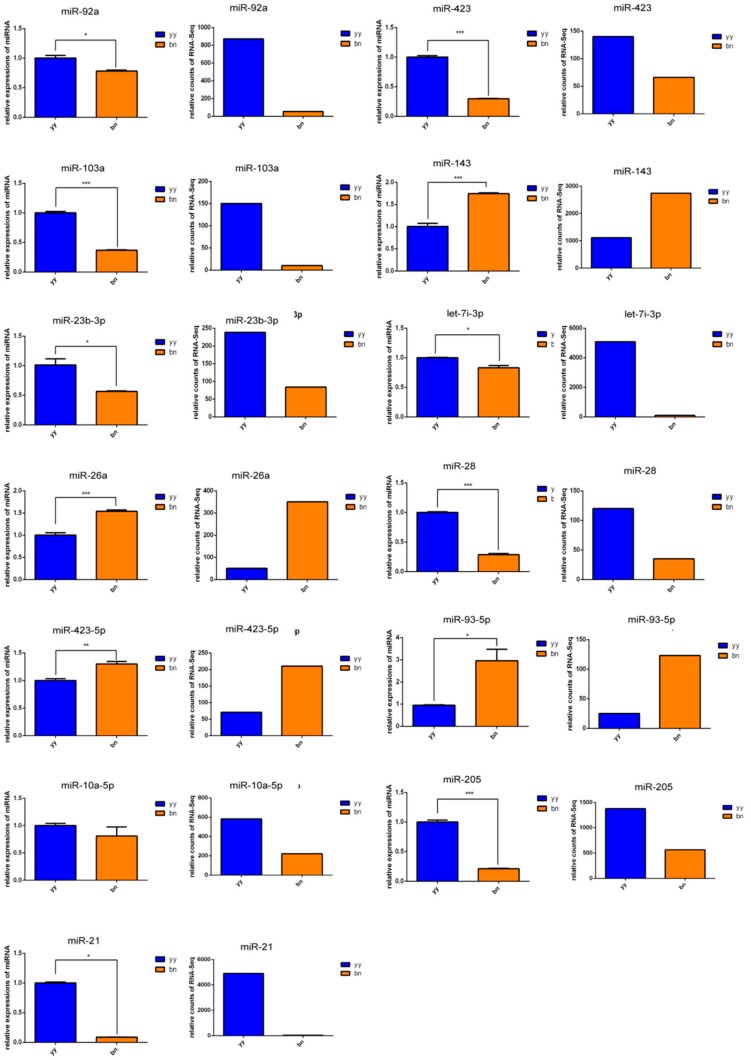
Validation of the HiSeq results through examining the expression levels of 13 DEMs by using qRT-PCR. U6 snRNA were used as reference genes. yy and bn indicated vegetative cells and dormant cysts of *P. cristata*, respectively. Error bars represent standard deviation of three independent experiments in duplicates. Significant differences compared to the control group are indicated with * *p* < 0.05; ** *p* < 0.01; *** *p* < 0.001.

Next, qRT-PCR analysis was performed to validate the HiSeq results of the DEMs. The identified DEMs such as miR-92a, miR-423, miR-103a, miR-143, miR-23b-3p, let-7i-5p, miR-26a, miR-28, miR-423-5p, miR-93-5p, miR-10a-5p, miR-205, and miR-21 were randomly selected for verifying their expression level in the dormant cysts and vegetative cells via qRT-PCR analysis. Our results showed that 12 DEMs in dormant cysts and vegetative cells of *P. cristata* were significantly consistent with the HiSeq results (Figure 4). Therefore, identification of DEMs in *P. cristata* was effective and credible. In addition, we performed a correlation analysis by using Excel correlation coefficient function, which assessed the validation of miRNA expression levels by qRT-PCR. The resulting correlation coefficient was 0.7323 (*R*^2^ = 0.7323). The correlation analysis further confirmed the reliability of the DEMs’ HiSeq results.

### 2.5. DEMs Target Gene Prediction and GO Annotation Analyses

Among the transcripts predicted from conserved DEMs, the most abundant target genes were ATP-dependent Clp protease ATP-binding subunit ClpB (clpB), glutamine synthetase (glnA), glutamine synthetase (glyS), chaperonin GroEL (groEL), heat shock 70kDa protein 8 (HSPA1_8), importin subunit beta-1 (IPO1), polyadenylate-binding protein (PABPC), alkyl hydroperoxide reductase subunit C (ahpC), large subunit ribosomal protein L2 (RP-L2), threonyl-tRNA synthetase (TARS), etc. In addition, the most abundant target genes of novel differential expressed miRNAs were chaperonin GroEL (HSPD1), RP-L2, ahpC, glnA, and molecular chaperone DnaK (dnaK), etc.

GO functional analysis showed that 165 miRNA with 1217 target genes were predicated by the miRanda algorithm. To investigate the function of the miRNA target genes, we performed GO term of the predicted miRNA targets. The target genes were annotated to a total of 1421 GO terms, 741 target genes were significantly changed in GO categories such as the biological process (BP), cellular component (CC), and molecular function (MF), while 1120 and 513 transcripts were predicted from conserved DEMs and novel DEMs, respectively.

**Figure 5:**
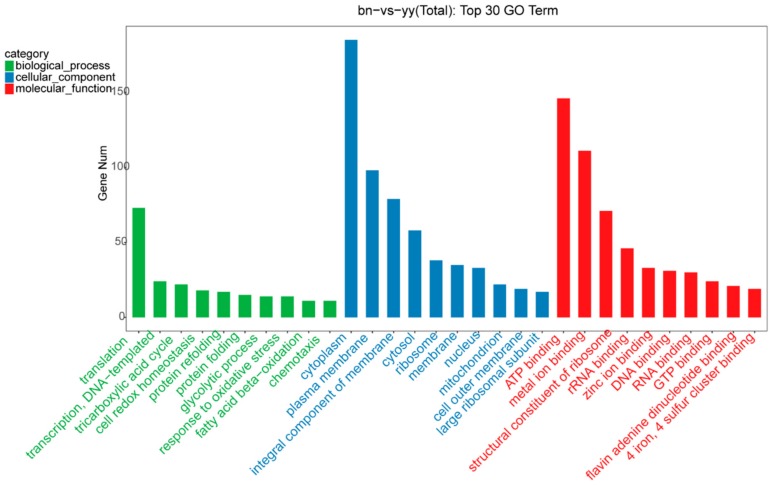
Top 30 miRNAs GO classification annotated for predicted target genes. GO classification included biological process (in green), cellular component (in blue), and molecular function (in red).

**Figure 6:**
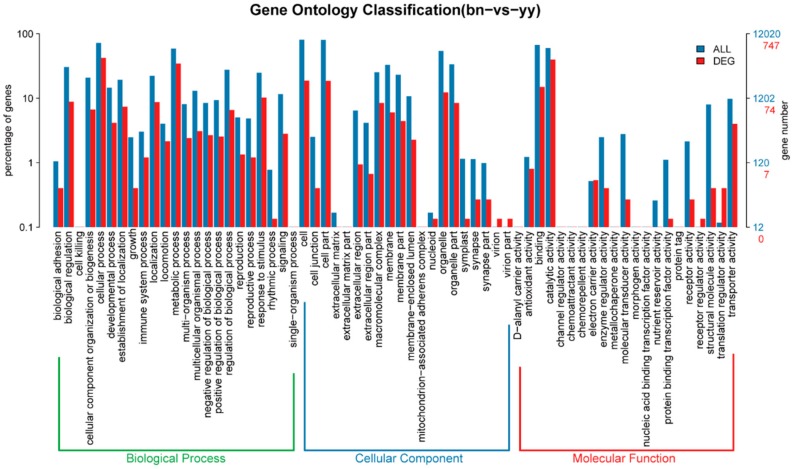
The distribution comparison of DEMs target genes and genes at GO Level2. Blue indicates all genes (ALL) entries at GO Level 2, red indicates the GO Level 2 entries enriched by DEMs target genes (DEG). The horizontal axis and the vertical axis are the entry name and the number of genes corresponding to the entry and its percentage.

Furthermore, the top 30 GO term annotation results showed that translation, cytoplasm, ATP binding, and metal ion binding were the most significantly enriched terms in both groups (Figure 5). The plasma membrane, integral component of membrane, and structural constituent of ribosome were also significantly enriched GO terms. A comparison of the differential distribution of miRNA target genes and all genes at the GO Level 2 was shown in Figure 6. The most enriched miRNA target genes in the GO BP category were the cellular process and the metabolic process with the number of genes under these entries were 315 and 257, respectively. The most enriched entries in the CC category were cell and cell part with the number of genes 140 and 139, respectively. The most enriched entries in the MF category were catalytic activity and binding with the number of genes under these entries were 297 and 112, respectively.

Next, we used TopGO directed acyclic graphs to visualize the GO nodes (terms) enriched by target genes and their hierarchical relationships. The directed acyclic graph of Term extracted by the target gene GO analysis was shown in Figure S1. The enriched BP related term was mainly GO: 0019752 carboxylic acid metabolic process; the enriched categories in CC was GO: 0030313 cell envelope and the enriched categories in MF was GO: 0016491 oxidoreductase activity.

### 2.6. The DEMs KEGG Functional Annotation Analyses

**Figure 7:**
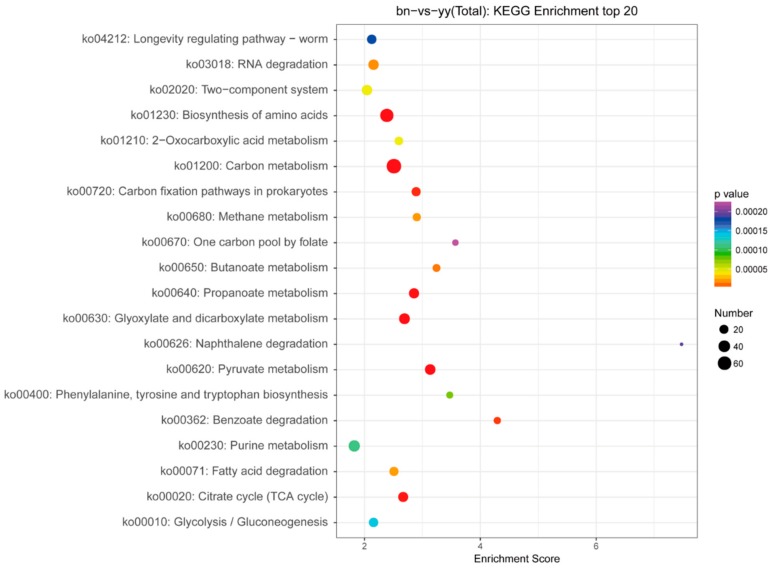
Top 20 bubble map of KEGG enrichment DEM target genes. The *x*-axis Enrichment Score is the enrichment score. The larger size bubble indicates a greater number of DEM target genes, and the smaller *p*-value means the greater significance.

KEGG pathway enrichment analysis was performed for the investigation of target mRNAs in the miRNA-mRNA pairs. The KEGG enrichment analysis of the target gene of DEMs showed that the total numbers of genes with KEGG pathway were 6048, and the number of genes with significant changes in the KEGG pathway were 486. KEGG analysis identified a total of 211 differentially expressed pathways in both groups, and the top 20 pathways associated with encystment were identified. Next, we created a bubble map of the top 20 KEGG pathway enrichment according to −log10 *p*-value corresponding to each entry (Figure 7).

**Figure 8:**
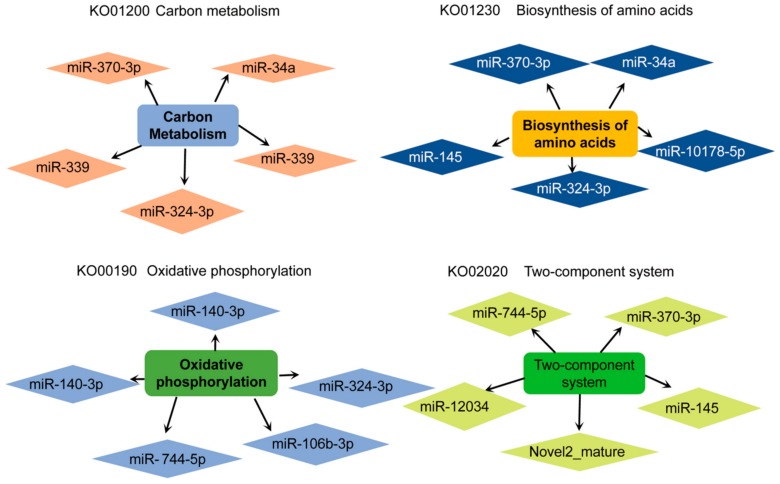
Co-expression networks among the important DEMs and the target mRNAs enriched for signaling pathways in the transformation of vegetative cells into dormant cysts of *P. cristata*. Diamonds represent the DEMs within each signaling pathway, and round rectangles represent the signaling pathways associated with relative target genes.

It was found that the signaling pathways regulated by miRNA were significantly enriched in the carbon metabolism (ko01200), biosynthesis of amino acids (ko01230), RNA degradation (ko03018), oxidative phosphorylation (ko00190), fatty acid degradation (ko00071) and two-component system (ko02020), etc. The co-expression networks among some of the important DEMs and the target mRNAs enriched for signaling pathways in the transformation of vegetative cells into dormant cysts of *P. cristata* were shown in Figure 8. Further details about KEGG Level 2 distribution map of DEMs target genes were represented in Table S8. Most KEGG pathway annotation in Level 2 highlighted the global and overview map, carbohydrate metabolism, translation, energy metabolism, signal transduction, folding, sorting and degradation, nucleotide metabolism, aging, and lipid metabolism.

### 2.7. DEMs Target Genes and Relative KEGG Signaling Pathways Involved in Encystment of P. cristata

To further identify the regulatory mechanisms involving miRNAs during the encystment, we focused on the relative miRNAs related closely to the encystment by bioinformatics analysis. We found that miRNAs were significantly downregulated in the encystment including miR-23b-3p, miR-370-3p, miR-28, miR-744-5p, miR-145, novel2_mature, miR-193b, and so on. However, the target gene of the downregulated miR-23b-3p is glutathione peroxidase (GPX), which was upregulated and played an important role in the ko00480 Glutathione metabolism signaling pathway. The downregulated miR-193b can negatively regulate its target gene aspartyl-tRNA synthetase (asps) and participate in the aminoacyl-tRNA biosynthesis (ko00970) signaling pathway. The downregulated miR-370-3p and miR-145 shared a common target gene, the upregulated glutamine synthetase (glnA), and glnA participated in the two-component system signal pathway (ko02020). In addition, downregulated miR-744-5p can upregulate ubiquinol-cytochrome c reductase iron-sulfur subunit (UQCRFS1) at oxidative phosphorylation (ko00190) and the two-component system signaling pathway (ko02020). The downregulated miR-28 can upregulate the target gene acyl-CoA oxidase (ACOX1) in the cAMP signaling pathway (ko04024). The downregulated miR-370-3p had two upregulated target genes threonyl-tRNA synthetase (TARS) and glycyl-tRNA synthetase alpha chain (glyS), which plays a critical role in an aminoacyl-tRNA biosynthesis signaling pathway. The downregulated novel2_mature can upregulate the target gene ubiquitin-activating enzyme E1 (UBE1), carnitine O-palmitoyltransferase 1 (CPT1), actin beta/gamma 1 (F-Actin), and molecular chaperone HtpG (HtpG). These target genes were involved in ubiquitin mediated proteolysis (ko04120), AMPK (ko04152) signaling pathway, regulation of actin cytoskeleton (ko04810), and PI3K-AKT (ko04151) signaling pathway, respectively. The downregulated miR-10178-5p regulates the upregulation of long-chain acyl-CoA synthetase (ACSL) in the fatty acid degradation (ko00071) signal pathway.

Significantly upregulated miRNAs were related to the encystment including miR-1307, miR-1180, miR-339, miR-106b-3p, and miR-143, etc. Encompassed by the upregulated miR-143 regulates the target gene serum/glucocorticoid-regulated kinase (SGK) downregulation in the FOXO (ko04068) signaling pathway. In addition, miR-1307, miR-1180, and miR-339 were upregulated and involved in the downregulation of RP-L2 in ribosome (ko03010). In addition, miR-1307 is also involved in the downregulation of rubredoxin-NAD^+^ reductase (rub) in the fatty acid degradation (ko00071) signal pathway. The upregulated miR-106b-3p was participated in the downregulation of the target gene cytochrome c oxidase subunit 5b (COX5B) in the oxidative phosphorylation (ko00190) signaling pathway.

Above all, differentially expressed genes such as GPX, UBE1, SGK, CPT1, and F-Actin were consistent with our previously reported findings [3]. According to the current investigation, we listed a miRNA–mRNA interaction relationship related to the encystment (Table 4). Our results indicated that these miRNAs and their potential target genes may have important regulatory roles in the transformation of vegetative cells into dormant cysts of *P. cristata*.

## 3. Discussion

Illumina HiSeq high-throughput sequencing is a powerful tool for the identification of differentially expressed genes, alternative splicing, and other genetic traits in various organisms [13,14]. In this study, we applied this method to identify and analyze the expression assessment of DEMs with the target genes in the dormant cysts compared with the vegetative cells in *P. cristata*. We identified total 449 DEMs between the dormant cysts and the vegetative cells, in which 243 DEMs were upregulated and 206 DEMs were downregulated in the dormant cysts. GO analysis indicated that target genes of DEMs were enriched in functions related to the cytoplasm. The previous morphological study found that, during encystment of *P. cristata*, cytoplasmic structures such as extrusomes were wrapped in membrane vesicles to form autophagic vesicles or autophagosomes in the intracellular self-digestion phenomenon [15]. Scientists found that knockdown of miR-23b-3p expression in LEC cells elevated autophagy significantly during hydrogen peroxide stress [16]. We found that miR-23b-3p was downregulated during the cyst formation. These facts suggested that miR-23b-3p downregulation may enhance autophagy, which promoted dramatic changes of the cell morphology and structure during the encystment. In addition, miR-23b-3p downregulation promoted the upregulation of the target gene GPX. GPX is an important antioxidant enzyme in organisms. It mainly scavenges organic peroxides, especially the large amount of lipid peroxides produced when free radicals cause lipid peroxidation. Moreover, GPX is also involved in a series of important biological processes such as the cell cycle, cell signal transduction, chromatin remodeling, DNA, and histone modification [17,18,19,20]. Therefore, the upregulation of GPX enhances the antioxidant and anti-stress capabilities of the cysts. These results suggested that downregulation of miR-23b-3p not only enhanced autophagy in the encystment of *P. cristata* but also improved the ability of the cysts to resist stress with increased GPX. These findings are consistent with our previous results [3].

Novel 2 mature was downregulated in the differential expressed, which is a new miRNA that upregulated several target genes such as F-Actin and CPT1. Previous studies found that F-Actin was involved in the regulation of cytoplasmic autophagy of *P. cristata* during the encystment [15]. Autophagy is a general strategy to survive adverse environmental conditions such as starvation condition [21]. Autophagy and autophagosomes were often observed in encysting ciliate cells and mature resting cysts [15].

Cell autophagy mainly functions as the maintenance of metabolic stability of matter and energy during starvation by removing abnormal or redundant structures in the cell, and cytoskeleton regulation involved various cellular processes such as membrane rearrangement and vesicle transport. Report suggested that intact F-actin filaments facilitates the formation of late-stage autophagosomes and degradation under normal or nutrient starvation conditions. Together, F-actin is required for both basal and starvation-induced autophagy in higher eukaryotes [22]. Therefore, F-actin upregulation could promote vegetative cell’s shrinkage of *P. cristata* and enhanced the cell autophagy via regulating actin cytoskeleton, thereby promoting the formation of the dormant cysts [9]. CPT1 is a key enzyme for β-oxidation of fatty acid, and increased expression of CPT1 causes decomposition of fatty acids [23]. It was suggested that upregulation of CPT1 could promote the β-oxidation of fatty acids in *P. cristata* and uses the ATP to support energy for the cyst survival.

As we know, miR-143 is a typical multifunctional miRNA that can weaken energy metabolism by inhibiting glycolysis [24]. We found that miR-143 was upregulated during the cyst formation, which suggested that miR-143 played an important role in the reduction of the cyst energy metabolism, which is beneficial for the cysts to enter the dormant state. In addition, our results from bioinformatics analysis showed that expression of miR-143 was inhibited by the regulation target genes such as SGK. SGK may be further regulated by variety of channels and transporters, such as epithelial sodium channel [25]. The downregulation of SGK could inhibit Pca cells growth significantly [26]. Therefore, we speculated that upregulated miR-143 could target the SGK and inhibited its expression, thereby inhibiting the activity of sodium channels and the growth and proliferation of vegetative cells in *P. cristata*, and meanwhile promoting the encystment.

As for miR-28, some studies showed that miR-28 was positively associated with cell death, overexpression of miR-28 played a detrimental role to cell survival, and the oxidative stress induced apoptosis was indeed rescued by an miR-28 antisense inhibitor [27]. In our study, it was revealed that the downregulation of miR-28 was beneficial for the cysts to survive in adversity. ACOX was the target gene of miR-28, which was a rate-limiting enzyme for the first step of the dehydrogenation of peroxisomal fatty acid β-oxidation [28]. Additionally, ACOX1 could inhibit fat accumulation [29], which suggested that miR-28 participated in the regulation of fatty acid metabolism of the encystment of *P. cristata* by upregulating ACOX, and provided the energy consumption for the cyst survival without the need to ingest food from the external environment.

Interestingly, our data also showed that downregulated miR-370-3p could upregulate target genes TARS and glyS, and downregulation of miRNA miR-193b could upregulate target genes asps. The target genes asps, TARS, and glyS were involved in tRNA aminoacylation for protein translation. They participated in protein synthesis of the Aminoacyl-tRNA biosynthesis and played an important role in the regulation of biosynthesis. Interestingly, TARS in lower creatures could recognize and combine with the tRNA clover structure simulated by its own mRNA to control the content of TARS in cells by blocking mRNA translation and affecting protein synthesis in an adversity, and then regulate cell metabolism to improve organism resistance to adversity [30]. Some studies had shown that glyS could act on extracellular regulated protein kinases (ERK) to dephosphorylate them, thereby inhibiting ERK signal transduction and preventing cell proliferation [31]. ERK played an important role in the suppression of cell proliferation. These facts suggested that the phenomenon of cells to stop proliferating and encystment of *P. cristata* may be related to miR-370-3p and miR-193b and their regulated target genes TARS, glyS, and asps.

Out of DEMs, the downregulated miR-324-3p had target gene citrate synthase (CS). The CS is a key enzyme of the citric acid cycle of mitochondria that provides energy for cellular function and plays a central metabolic role in aerobics and other organisms [32,33]. Because of these functions, the CS might be involved in the regulation of energy supply required for the cyst formation. In addition, we speculated that the upregulation of CS during the encystment of *P. cristata* was in response to adversities’ conditions, which played a vital role in various redox reactions of the organism.

Bioinformatics results showed that the upregulated miR-106b-3p regulated the downregulation of COX5B in mitochondria. COX5B is a terminal enzyme of the electron transport chain, which transfers electrons from reduced cytochrome c to oxygen. In this process, an electrochemical gradient is generated on the inner mitochondrial membrane [34]. Literature also reported that the activity of cytochrome c oxidase was an important control point in the overall regulation of cellular energy metabolism [35]. Therefore, the downregulation of COX5B expression suggests that mitochondrial energy supply was weakened during the cyst formation, and coordination of the cysts into a low-energy metabolic state was conducive to survival in adversity. The literature also documented that the absence of COX5B results in cell growth inhibition. It is therefore indicated that downregulating COX5B might have a role in inhibiting the cell growth and promote the cyst formation of *P. cristata*.

Furthermore, the literature had also reported that the two-component system is widespread in a variety of lower eukaryotes, prokaryotes, and plants, indicating the regulation of signaling pathways in prokaryotes and eukaryotes [36]. Our RNA-seq results also revealed that miR-12304, miR-744-5p, and miR-145 controlled the upregulation of high temperature requirement protease A (HtrA), UQCRFS1, and GlnA. HtrA, also known as DegP, is a membrane serine protease with heat shock protein properties [37]. DegP also exhibits ATP-independent protease activity and plays an important role in the removal of toxic protein misfolding in the periplasmic space of the cell. The enzymatic activity of DegP is upregulated by various environmental stresses, such as heat shock, oxidative stress, and the presence of reducing agents [38]. Our data showed that miR-12304 regulated HtrA/DegP upregulated during the encystment, suggesting that miR-12304 controlled protein quality by upregulating HtrA/DegP during the encystment to promote the breakdown of misfolded proteins, and also coping with stress conditions, enhancing the cyst survivability in adversity. UQCRFS1, otherwise known as the Rieske Fe-S protein, is a key subunit of the cytochrome bc1 complex (complex III) in the mitochondria. The Rieske Fe-S protein is important in redox reactions in the mitochondria as it is involved in electron transport [39]. Therefore, during the process of *P. cristata* encystment, the amplification/variation of this gene suggests that the mitochondrial respiratory chain is involved in the encystment of *P. cristata*.

GlnA, also known as GS, catalyzes the ATP dependent ligation of glutamate and ammonia to synthesize glutamine [40]. GS is crucial during the maintenance of the glutamate–glutamine cycle [41]. During the stress conditions, the ammonia concentration usually raises in the tissues due to more active catabolism of amino acid and proteins that confronts the host with endogenous ammonia toxicity. Therefore, the excess ammonia must be either pumped out of the body by immersion or converted into a toxic Gln [42,43]. A previous report found that the GS gene has osmo-regulation properties in the osmotic stress environments. Moreover, GS participated in osmo-regulating the toxic ammonia and increased the Gln levels in the tissues [44]. Furthermore, the mRNA expression was significantly upregulated in the *P. cristata* during the osmotic challenge in the encystment. Therefore, we speculated that GS may be involved in a variety of biological processes such as homeostasis and cell signaling regulation in the encystment of *P. cristata*.

**Figure 9:**
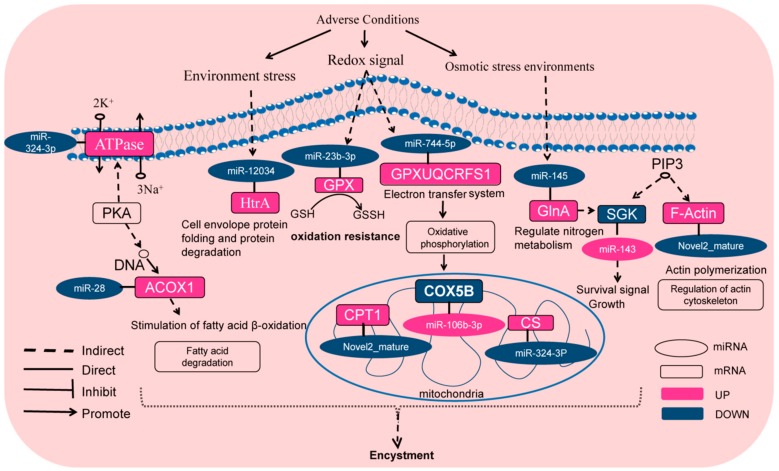
Schematic diagram of hypothetical signaling network of miRNAs regulating or promoting *P. cristata* encystment. Pink color indicates upregulation and blue color represent downregulation of gene expression during the encystment of *P. cristata*.

Taken together, the analysis of these DEMs involved in the encystment indicated that the DEM target genes highlighted the pathways of not only two-component system, but also AMPK, cAMP, and PI3K-AKT signaling pathway through the biological processes. Based on the above results, we have proposed a schematic diagram of a hypothetical signaling network of miRNAs regulating or promoting *P. cristata* encystment (Figure 9). In response to the adverse environment stress, some important DEMs including miR-28, miR-23b-3p, miR-12034, miR-744-5p, miR-145, miR-143, miR-106b-3p, novel 2 mature, etc. participate in the promotion or regulation of the encystment by regulating their target gene and signaling pathways, which may be contributed to the encystment of *P. cristata*.

## 4. Materials and Methods

### 4.1. Cell Culture and the Encystment Induction

The *P. cristata* (Ciliophora, Urostylida) was provided by the Professor Fukang Gu from East China Normal University. The *P. cristata* specimens were collected from paddy-field water in Southern Anhui, China, in May, 2005. The ciliate cells were cultured in 10 cm Petri dishes with treated pond water (ZiZhuyuan in East China Normal University, Shanghai) and incubated at 25 °C. The pond water was filtered by Tzakzy paper which was produced from cotton linters and treated by an autoclave (LDZF-75L, Shanghai Shenan Co, Ltd., China) for 30 min at 121 °C. 1 or 2 boiled wheat grains were added into the culture to enrich bacteria as the food of ciliates cells. When the cultured vegetative cells reached a high density, the wheat grains were removed and most vegetative cells formed the cysts within 3–4 days.

### 4.2. Sample Collection and RNA Isolation

For RNA extraction, approximately 2 × 10^4^ cells were collected and transferred into 1.5 mL centrifuge tube, the cells were centrifuged at room temperature for 2 min at 3000 rpm, and then total RNA was extracted using mirVana™ miRNA ISOlation Kit (Ambion-1561) according to the manufacturer’s guideline. The quality of RNA was determined using a NanoDrop and Agilent 2100 (Agilent Technologies, Inc., USA). The vegetative cells (yy) were used as the control group and the induced cysts (bn) were used as the experimental group.

### 4.3. Small RNA Library Construction and Sequencing

Total RNA was ligated with 3′ and 5′ adapters. Reverse transcription PCR was performed using the reverse transcription primers. The PCR reactions were conducted using forward and reverse primers and then the quality of DNA was tested with the High Sensitivity DNA Chip. Purify cDNA was constructed by RNA Gel Electrophoresis. Afterwards, a library quality test was done by adding 1 µL samples to Agilent 2100 chip to confirm the length and quality of library. Finally, the qualified cDNA library was sequenced using the Illumina HiSeq X Ten sequencing platform. Sequence Read Archive (SRA) databases have been submitted under accession numbers SRR10854670 and SRR10854669.

### 4.4. Sequence Analysis and Identification of miRNAs

The raw reads were processed by evaluating the sequencing quality, calculating the length and distribution of small RNA reads, and removing adaptor sequences and low-quality reads such as sequences with low Q20. Clean and high-quality reads with lengths of 15–41 nt were used in the subsequent analyses. To analyze the distribution of small RNAs based on the reference sequence, the clean sequencing reads were mapped to the small RNA databases using Bowtie [45]. There was a wide variety of small RNAs, including miRNAs, tRNAs (tiRNAs, tRFs), rRNAs, piRNAs, snRNAs, and more. In order to classify and annotate small RNA in sequencing results, clean reads were sequentially compared and annotated with the Reference genome, Rfam database [46], cDNA sequence, species repeat sequence library [47], and miRBase database [48,49].

First of all, these clean sequences were mapped onto the reference genome. Then, the unique reads were compared with the Rfam database (version 10.0). The sequence annotation results of rRNA, snRNA, Cis-reg, tRNA, and other Rfam RNA were finally filtered out. They were not used for subsequent known miRNA alignments and new miRNA predictions. Extracting a sequence with a sequence length of 15–26 nt by transcript sequence alignment used for subsequent known miRNA alignments and new miRNA predictions. After that, comparing against the reads with Repbase database by RepeatMasker software (version open-4.0.7) identified possible repeats and then filtered them. At last, bowtie software was used to perform error-free alignment with the miRNA mature sequence in miRBase to get the details of miRNA in *P. cristata*. Through the comparison of the above databases, the distribution of small RNA in each sample was finally summarized.

The remaining unannotated sRNA sequences were used for novel miRNA Prediction. The identification of novel miRNAs was performed using miRDeep2 [50] and RNAfold [51]. The miRDeep2 software was used to align the unannotation reads with the genomic sequence, provided that the sequence was at least 18 nt in length and capable of mapping the genome. The RNAfold software was used to predict the secondary structure of the sequence capable of mapping the genome, and the sequence capable of forming the miRNA hairpin precursor was considered to be a new miRNA sequence.

### 4.5. MiRNA Differential Expression Analysis

The expression levels of miRNAs in the different libraries constructed from *P. cristata* were estimated based on the Illumina sequencing data according to the TPM clean reads [52]. The normalized expression values were calculated using the following formula: (read count × 10^6^) / total miRNA read count in the library. The *p*-value was calculated using the Audic_Claverie formula [53]. In brief, TPM fold change > 2 and *p*-value < 0.05 were used as the cut-off criteria of the significant DEMs between the dormant cysts and vegetative cells.

### 4.6. Prediction of miRNA Targets Gene, GO Enrichment, and KEGG Pathway Analysis

The potential target genes of the miRNAs were predicted using miRanda (http://www.microrna.org/microrna/home.do/) according to the default parameters of the sofware [54]. The KEGG and GO analysis of the target genes were performed using the hypergeometric distribution test of the R software. Moreover, the *p*-value was corrected by the Benjamini and Hochberg multiple tests to obtain the false discovery rate (FDR). The target genes of DEM pathways were used to look for the role of DEMs signaling in the encystment of *P. cristata*.

### 4.7. qRT-PCR Analysis

In order to verify the accuracy of high throughput sequencing results, we randomly selected and confirmed the expression of 13 DEMs in the vegetative cells and the dormant cysts by qRT-PCR. Total RNA was extracted using the MiniBEST Universal RNA Extraction Kit (TaKaRa, Shanghai, China). The total RNA was transcribed into cDNA by using Mir-X™ miRNA First Strand Synthesis Kit (TaKaRa, Shanghai, China) according to the manufacturer’s instructions. The primers were designed for qRT-PCR verification by Sangon Biotech (Shanghai, China). The volume of qRT-PCR reaction mixture was 20 µL consisting of 10 µL TB Green Premix Ex Taq II (Tli RNaseH Plus), 0.8 µL mRQ 3’ primer (10 µM), 0.8 µL specific primer (10 µmol/L), 0.4 µL ROX Reference Dye II, 4 µL cDNA, and 4 µL double-distilled H_2_O. The amplification reactions were incubated at 95 °C for 30 s, followed by 40 cycles of 95 °C for 5 s, and 60 °C for 30 s. The reaction of qRT-PCR was performed with a QuantStudio 3 instrument (Thermo Fisher Scientific, USA). The 5′ to 3′ specific primers of these genes are shown in Table S9. All of the reactions were repeated in triplicate and the relative expression levels were calculated using the 2^−^^ΔΔCt^ method [55]. U6 snRNA was used as internal control.

### 4.8. Data Analysis

Statistical analysis of the transcriptomic data was described in the corresponding sections. Group comparison was done with independent *t*-tests. *p* < 0.05 was considered to be statistically significant.

## 5. Conclusions

In summary, we have identified a large number of miRNAs from vegetative cells and dormant cysts of *P. cristata* and also analyzed their expression pattern in different states and predicted their putative targets. Furthermore, DEMs in the cyst were identified in comparison to the vegetative cells. A total 449 unique DEMs were identified, of which 243 DEMs were upregulated and 206 DEMs were downregulated. Among them, 94 were specifically expressed in the vegetative cells and 83 expressed in the dormant cysts. Additionally, the DEMs in two-component systems, AMPK, cAMP, and PI3K-AKT signaling pathways, which might participate in the regulation process of *P. cristata* encystment. Furthermore, it will be very important to experimentally characterize these DEMs and downstream targets will lead to a better understanding of their functional relationships and mechanisms within the regulatory network. However, the role of these DEMs in the formation of cysts of *P. cristata* remains to be further verified.

## Figures and Tables

**Table 1:** Clean reads statistics in the libraries of vegetative cells and dormant cysts.

Sample	Raw Reads	Reads Trimmed Length	Reads Trimmed Q20	Reads Trimmed N	Clean Reads	Clean Reads Uniq
bn	33,344,602	28,940,389	28,913,796	28,853,904	28,853,904	1,570,219
yy	31,646,444	30,456,518	30,434,734	30,368,742	30,368,742	844,821

The dormant cysts are indicated as bn, whereas vegetative cells are indicated as yy.

**Table 2:** Distribution of reads in the libraries of vegetative cells (yy) and dormant cysts (bn).

Sample	Annotation Type	Number of Total	% of Total	Number of Uniq	% of Uniq
yy	rRNA	2504	0.01%	670	0.08%
	tRNA	270	0.00%	124	0.01%
	snRNA	748	0.00%	397	0.05%
	Cis-reg	722	0.00%	391	0.05%
	other_Rfam_RNA	2593	0.01%	764	0.09%
	gene	703,473	2.32%	31,193	3.69%
	repeat	870,399	2.87%	38,129	4.51%
	known_miRNA	178,799	0.59%	2186	0.26%
	unannotation	28,609,234	94.21%	770,967	91.26%
bn	rRNA	16234	0.06%	889	0.06%
	tRNA	775	0.00%	169	0.01%
	snRNA	6346	0.02%	693	0.04%
	Cis-reg	3169	0.01%	686	0.04%
	other_Rfam_RNA	7899	0.03%	1366	0.09%
	gene	1,737,462	6.02%	52,551	3.35%
	repeat	4,173,497	14.46%	56,926	3.63%
	known_miRNA	238,353	0.83%	2462	0.16%
	unannotation	22,670,169	78.57%	1,454,477	92.63%

Annotation type: annotation category. Number of total: the number of corresponding annotation categories in the total reads of the sample. % of total: the proportion of corresponding annotation categories in the total reads of the sample. Number of unique: the number of corresponding annotation categories in the sample uniq reads. % of unique: the proportion of corresponding annotation categories in the sample uniq reads.

**Table 3:** List of novel miRNAs identified in *P. cristata*.

Novel ID	MiRDeep2 Score	Consensus Mature Sequence	Consensus Precursor Sequence	Precursor Coordinate
novel1	6.2	cggcucgauuuguuugaaauac	guuucacucaucaucgagcuguuggacggcucgauuuguuugaaauac	TRINITY_DN19462_c0_g1_i2:301…349:+
novel2	4.4	ccggcggcggcgaucaugaagugc	ccggcggcggcgaucaugaagugcuucggcauggaauguuuucgucgucgcuaguucgg	TRINITY_DN23739_c0_g5_i2:245…304:+
novel3	4.2	uaacggaaacaacgaucagcc	cugauggaggugaagguaaugguggauauaacggaaacaacgaucagcc	TRINITY_DN14496_c0_g2_i1:253…302:+
novel4	2.1	cggcgcgccgggcccggc	cagcccggcgugcagggcccccagggcccgccgggauacccuggcgacaugggccccgugggccgcaccggcgcgccgggcccggc	TRINITY_DN14556_c0_g1_i1:557…643:+
novel5	1.1	uuuauuucgguaugucugc	aagcauauugaacuuuacaauuaaauucuggguguccucuguggugaggauuucugaguaaauaccucuuuauuucgguaugucugc	TRINITY_DN17506_c0_g1_i1:476…563:+
novel6	0.9	aaggcugaaacuuaaagga	cuuuaaguuaggcuuugcuaauaaaggcugaaacuuaaagga	TRINITY_DN24369_c0_g1_i1:2878…2920:-
novel7	0.9	uggacggcgggguccuugcggac	uggacggcgggguccuugcggacuuccccagcuucggucggggaagucggaugagacuucggcggucuuacg	TRINITY_DN23739_c0_g3_i2:205…277:+
novel8	0.5	ucgguaucaacgaacuccuuga	cacgcggggaguugcaaugaauccaaucgaucauccacacggaggacgaacgaaagcgguucgguaucaacgaacuccuuga	TRINITY_DN24930_c0_g1_i1:681…763:+

**Table 4:** Potential role of miRNAs and selected target genes in the encystment of *P. cristata*.

No.	Target Gene Symbol	Gene Description	Biological Processes	Up Down	Related Identified miRNAs
1	GPX	glutathione peroxidase	glutathione peroxidase activity	up	miR-23b-3p↓
2	asps	aspartyl-tRNA synthetase	tRNA aminoacylation for protein translation	up	miR-193b↓
3	TARS	threonyl-tRNA synthetase	tRNA aminoacylation for protein translation	up	miR-370-3p↓
4	glyS	glycyl-tRNA synthetase alpha chain	arginyl-tRNA aminoacylation	up	miR-370-3p↓
5	glnA	glutamine synthetase	putrescine binding	up	miR-370-3p↓, miR-145↓
6	UQCRFS1	ubiquinol-cytochrome c reductase iron-sulfur subunit	ubiquinol-cytochrome-c reductase activity	up	miR-744-5p↓
7	ACOX1	acyl-CoA oxidase	very long-chain fatty acid metabolic process	up	miR-28↓
8	UBE1	ubiquitin-activating enzyme E1	ubiquitin activating enzyme activity	up	novel2_mature↓
9	CPT1	carnitine O-palmitoyltransferase 1	long-chain fatty acid metabolic process	up	novel2_mature↓
10	F-Actin	actin beta/gamma 1	structural constituent of cytoskeleton	up	novel2_mature↓
11	HtpG	molecular chaperone HtpG	protein folding	up	novel2_mature↓
12	ACSL	long-chain acyl-CoA synthetase	long-chain fatty acid-CoA ligase activity	up	miR-10178-5p↓
13	SGK	serum/glucocorticoid-regulated kinase	neuron projection morphogenesis	down	miR-143↑
14	RP-L2	large subunit ribosomal protein L2	structural constituent of ribosome; translation	down	miR-1307↑, miR-1180↑, miR-339↑
15	rub	Rubredoxin-NAD^+^ reductase	rubredoxin-NADP reductase activity	down	miR-1307↑
16	COX5B	cytochrome c oxidase subunit 5b	cytochrome-c oxidase activity	down	miR-106b-3p↑

## Supplementary Materials

Supplementary materials can be found at the file at https://www.mdpi.com/1422-0067/21/7/2309/s1. Table S1. Rfam database comparison statistics. Table S2. mRNA sequence alignment statistics. Table S3. Repbase alignment statistics. Table S4. Known miRNA sequence alignment statistics. Table S5. Known miRNA categories. Table S6. The top 20 abundant miRNAs. Table S7. The top 30 miRNA gene families. Table S8. KEGG Level 2 distribution map of DEMs target genes. Table S9. Primer sequences of real-time PCR for validation of the 13 miRNAs by quantitative RT-PCR. Figure S1. The topGO directed acyclic graph.

## Abbreviations

miRNAome	microRNAome
P. cristata	Pseudourostyla cristata
miRNAs	microRNAs
GO	Gene Ontology
KEGG	Kyoto Encyclopedia of Genes and Genomes
yy	vegetative cells
bn	dormant cysts
DEMs	differentially expressed miRNAs
BP	biological process
CC	cellular component
MF	molecular function
clpB	Clp protease ATP-binding subunit ClpB
glnA	glutamine synthetase
glyS	glycyl-tRNA synthetase alpha chain
groEL	chaperonin GroEL
HSPA1_8	heat shock 70kDa protein 8
IPO1	importin subunit beta-1
PABPC	polyadenylate-binding protein
ahpC	alkyl hydroperoxide reductase subunit C
RP-L2	large subunit ribosomal protein L2
TARS	threonyl-tRNA synthetase
HSPD1	chaperonin GroEL
dnaK	molecular chaperone DnaK
GPX	glutathione peroxidase
UQCRFS1	ubiquinol-cytochrome c reductase iron-sulfur subunit
ACOX1	acyl-CoA oxidase
TARS	threonyl-tRNA synthetase
UBE1	ubiquitin-activating enzyme E1
CPT1	carnitine O-palmitoyltransferase 1
F-Actin	actin beta/gamma 1
HtpG	molecular chaperone HtpG
ACSL	long-chain acyl-CoA synthetase
SGK	serum / glucocorticoid-regulated kinase
rub	rubredoxin-NAD^+^ reductase
COX5B	cytochrome c oxidase subunit 5b
asps	aspartyl-tRNA synthetase
qRT-PCR	quantitative real-time PCR
