# Supplementary material for: The Encystment-Related MicroRNAs and Its Regulation Molecular Mechanism in Pseudourostyla cristata Revealed by High Throughput Small RNA Sequencing
Source: Int J Mol Sci. 2020 Mar 26;21(7):2309. doi: 10.3390/ijms21072309 (PMC7177753; doi:10.3390/ijms21072309)
Supplement: Supplementary file 1 [file ijms-21-02309-s001.pdf]

International Journal of *Molecular Sciences*

MDPI

Supplementary Materials: The Encystment-Related MicroRNAs and Its Regulation Molecular Mechanism in *Pseudourostyla cristata* Revealed by High Throughput Small RNA Sequencing

Nan Pan*, Muhammad Zeeshan Bhatti*, Haiyang Zhang, Bing Ni, Xinpeng Fan* and Jiwu Chen*

Table S1. Rfam database comparison statistics.

Sample	Rfam_Type	Number_of_Total	%_of_Total	Number_of_Unique	%_of_Unique
	rRNA	2504	0.01%	670	0.08%
	tRNA	270	0.00%	124	0.01%
yy	snRNA	748	0.00%	397	0.05%
	Cis-reg	722	0.00%	391	0.05%
	other_Rfam_RNA	2593	0.01%	764	0.09%
	rRNA	16234	0.06%	889	0.06%
	tRNA	775	0.00%	169	0.01%
bn	snRNA	6346	0.02%	693	0.04%
	Cis-reg	3169	0.01%	686	0.04%
	other_Rfam_RNA	7899	0.03%	1366	0.09%

Table S2. mRNA sequence alignment statistics.

Sample	Reads	Aligned_Reads	Aligned(%)
yy	30368742	703473	2.32%
yy.uniq	844821	31193	3.69%
bn	28853904	1737462	6.02%
bn.uniq	1570219	52551	3.35%

Table S3. Repbase alignment statistics.

Sample	Clean_Reads	Aligned_Reads	Aligned(%)
yy	30368742	870399	2.87%
yy.uniq	844821	38129	4.51%
bn	28853904	4173497	14.46%
bn.uniq	1570219	56926	3.63%

Table S4. Known miRNA sequence alignment statistics.

Sample	Reads	Aligned_Reads	Aligned(%)
yy	30368742	178799	0.59
yy.uniq	844821	2186	0.26
bn	28853904	238353	0.83
bn.uniq	1570219	2462	0.16

Table S5. Known miRNA categories.

Samples	Known.miRNA.Categories
yy	1160
bn	1271

Table S6. The top 20 abundant miRNAs.

miRNA_id	yy.counts	miRNA_id	bn.counts
miR-26c	14648	miR-26c	20142
miR-148a	9326	let-7a	15537
let-7a	9209	miR-148a-3p	11868
miR-21-5p	7439	miR-21-5p	10711
miR-99b	6618	let-7f	9604
let-7f	5670	miR-100-5p	7035
let-7i-5p	5082	miR-99a-5p	6880
miR-21	4908	let-7i-5p	6856
miR-203	4725	miR-199-3p	5895
let-7g	4706	let-7g	5841
miR-100-5p	4667	miR-27b	5701
miR-451-5p	4516	miR-451	5680
miR-27b	4308	miR-21-5p	5197
miR-199-3p	4280	miR-203	4467
let-7b	3796	miR-100-5p	4396
miR-27a-3p	3194	let-7b	3990
let-7-5p	2839	miR-205	3985
miR-126	2641	miR-21-5p	3738
miR-100-5p	2100	miR-27a	3570
miR-205	1957	miR-126-3p	3447

Table S7. The top 30 miRNA gene families.

miFam_id	bn.counts	miFam_id	yy.counts
let-7	51333	let-7	36277
mir-10	30249	mir-10	21513
mir-26	23805	mir-26	18461
mir-21	19810	mir-21	14196
mir-27	14741	mir-27	11251
mir-148	12355	mir-148	9728
mir-199	9119	mir-203	6703
mir-203	8264	mir-3604	5794
mir-451	6867	mir-451	5478
mir-30	5502	mir-30	3964
mir-143	5154	mir-126	3951
mir-126	5116	mir-143	3835
mir-205	4622	mir-205	3386
mir-221	3349	mir-3596	3291
mir-146	3000	mir-199	2760
mir-23	2955	mir-23	2256
mir-8	2705	mir-221	2206
mir-3074	2534	mir-8	2148
mir-3556	1719	mir-3074	1965
mir-3604	1576	mir-146	1734
mir-363	1380	mir-3556	1267
mir-3596	1253	mir-363	969
mir-181	1143	mir-181	862
mir-25	1112	mir-25	792
mir-150	1005	mir-22	731
mir-191	968	mir-28	723
mir-28	961	mir-191	718
mir-378	947	mir-150	708
mir-22	944	mir-378	652
mir-423	840	mir-423	639

Table S8. KEGG Level 2 distribution map of DEMs target genes.

Classification_level2	Classification_level1	gene_number	percentage
Global and overview maps	Metabolism	121	24.9
Carbohydrate metabolism	Metabolism	97	19.96
Translation	Genetic Information Processing	96	19.75
Amino acid metabolism	Metabolism	88	18.11
Energy metabolism	Metabolism	75	15.43
Signal transduction	Environmental Information Processing	60	12.35
Folding, sorting and degradation	Genetic Information Processing	48	9.88
Nucleotide metabolism	Metabolism	43	8.85
Aging	Organismal Systems	35	7.2
Lipid metabolism	Metabolism	32	6.58
Metabolism of cofactors and vitamins	Metabolism	30	6.17
Metabolism of other amino acids	Metabolism	26	5.35
Xenobiotics biodegradation and metabolism	Metabolism	25	5.14
Cell growth and death	Cellular Processes	24	4.94
Endocrine system	Organismal Systems	24	4.94
Transport and catabolism	Cellular Processes	23	4.73
Membrane transport	Environmental Information Processing	23	4.73
Cell motility	Cellular Processes	19	3.91
Cellular community - prokaryotes	Cellular Processes	13	2.67
Transcription	Genetic Information Processing	13	2.67
Metabolism of terpenoids and polyketides	Metabolism	12	2.47
Immune system	Organismal Systems	11	2.26
Nervous system	Organismal Systems	11	2.26
Replication and repair	Genetic Information Processing	7	1.44
Biosynthesis of other secondary metabolites	Metabolism	7	1.44
Glycan biosynthesis and metabolism	Metabolism	7	1.44
Digestive system	Organismal Systems	7	1.44
Excretory system	Organismal Systems	6	1.23
Cellular community - eukaryotes	Cellular Processes	4	0.82
Circulatory system	Organismal Systems	4	0.82
Sensory system	Organismal Systems	3	0.62
Environmental adaptation	Organismal Systems	2	0.41
Signaling molecules and interaction	Environmental Information Processing	1	0.21
Development	Organismal Systems	1	0.21

Table S9. Primer sequences of real-time PCR for validation of the miRNAs by quantitative RT-PCR.

miRNA Primer ID	Sequence (5' to 3')
miR-92a	TATTGCACTTGTCCCGGCCTG
miR-423	TGAGGGGCAGAGAGCGAG
miR-103a	AGCAGCATTGTACAGGGCTATGAAG
miR-143	GGTCTGAGATGAAGCACTGTAGCTC
miR-23b-3p	CATCACATTGCCAGGGATTACCACA
let-7i-5p	CGCCTGAGGTAGTAGTTTGTGCTGTT
miR-26a	CGTTCAAGTAATCCAGGATAGGCTGTG
miR-28	GCACTAGATTGTGAGCTCCTGGAG
miR-423-5p	TGAGGGGCAGAGAGCGAG
miR-93-5p	CCAAAGTGCTGTTCGTGCAGGTAG
miR-10a-5p	CGCTACCCTGTAGATCCGAATTTGTG
miR-205	TCCTTCATTCCACCGGAGTCTG
miR-21	ACGCTAGCTTATCAGACTGATGTTGACTG

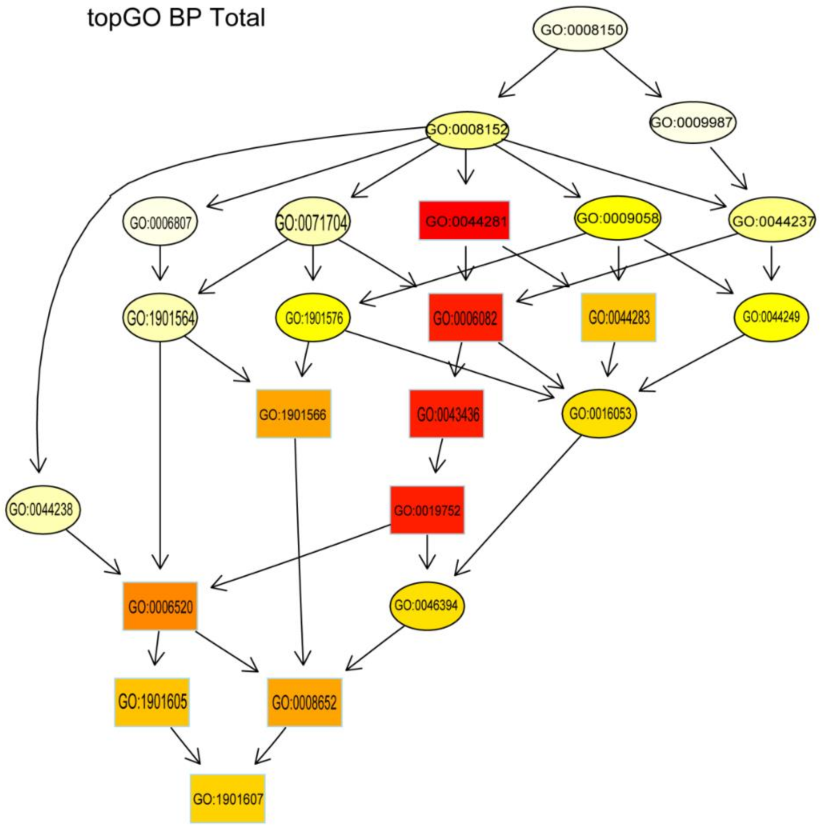
topGO BP Total
GO:0008150
GO:0008152
GO:0009987
GO:0006807
GO:0071704
GO:0044281
GO:0009058
GO:0044237
GO:1901564
GO:1901576
GO:0006082
GO:0044283
GO:0044249
GO:1901566
GO:0043436
GO:0016053
GO:0044238
GO:0019752
GO:0006520
GO:0046394
GO:1901605
GO:0008652
GO:1901607

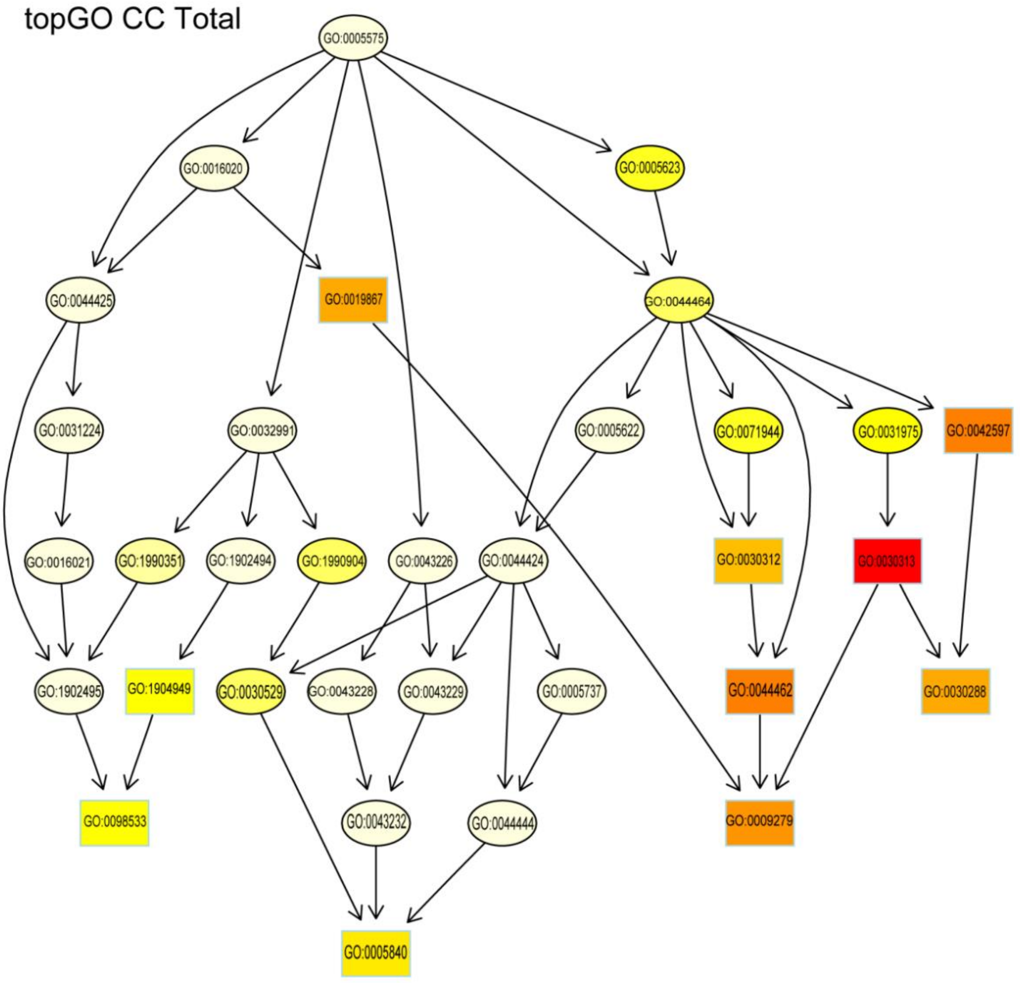
topGO CC Total
GO:0005575
GO:0016020
GO:0005623
GO:0044425
GO:0019867
GO:0044464
GO:0031224
GO:0032991
GO:0005622
GO:0071944
GO:0031975
GO:0042597
GO:0016021
GO:1990351
GO:1902494
GO:1990904
GO:0043226
GO:0044424
GO:0030312
GO:0030313
GO:1902495
GO:1904949
GO:0030529
GO:0043228
GO:0043229
GO:0005737
GO:0044462
GO:0030288
GO:0098533
GO:0043232
GO:0044444
GO:0009279
GO:0005840

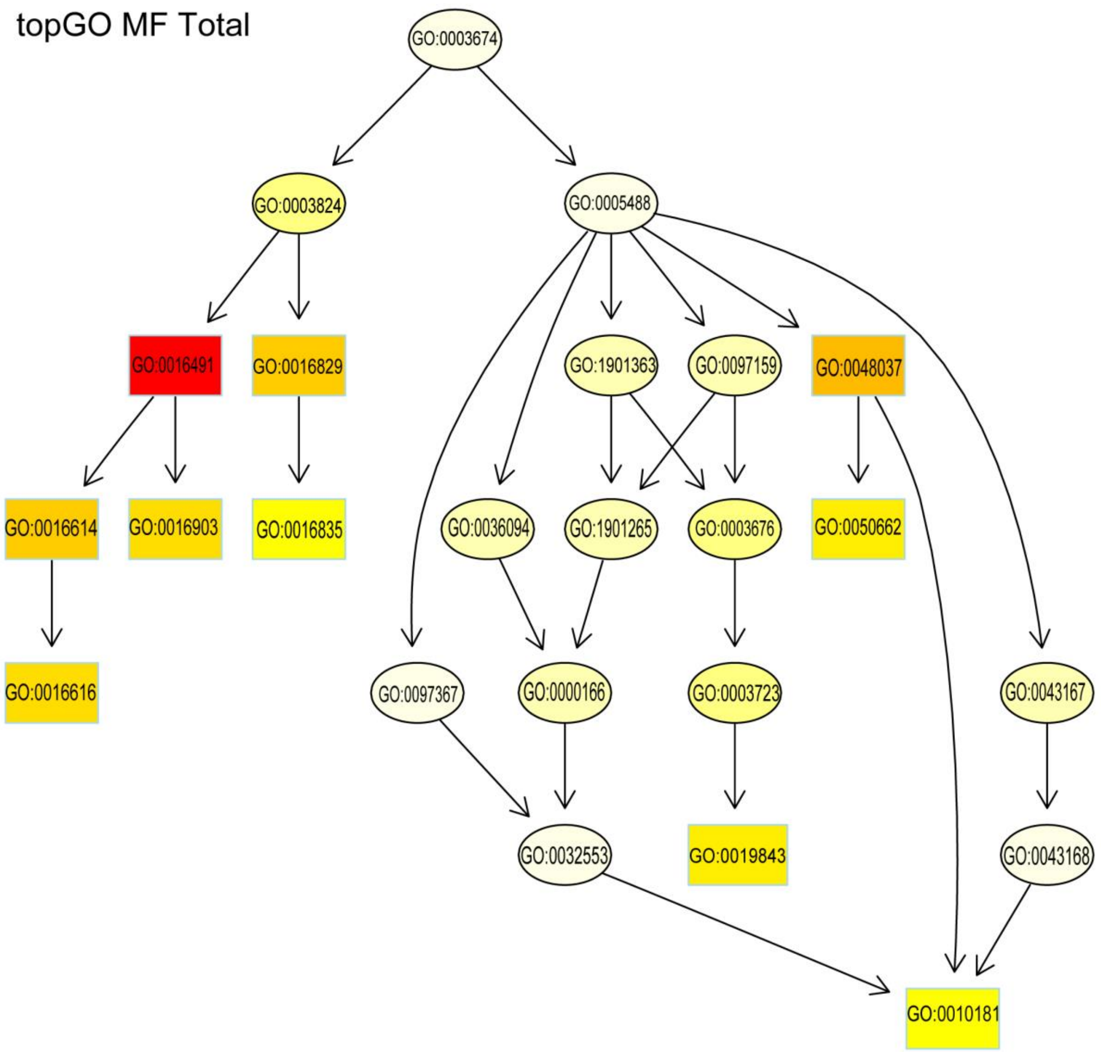

Figure S1. The topGO directed acyclic graph. The topGO directed acyclic graph visualizes the differential expression of GO nodes (Term) and their hierarchical relationships when the vegetative cells were transformed into dormant cysts. The enriched BP related term was mainly GO:0019752 carboxylic acid metabolic process; the enriched categories in CC was GO: 0030313 cell envelope; the enriched categories in MF was GO:0016491 oxidoreductase activity.
